# Understanding the Prevalence and Associated Factors of Behavioral Intention of COVID-19 Vaccination Under Specific Scenarios Combining Effectiveness, Safety, and Cost in the Hong Kong Chinese General Population

**DOI:** 10.34172/ijhpm.2021.02

**Published:** 2021-01-09

**Authors:** Yanqiu Yu, Joseph T.F. Lau, Mason M.C. Lau, Martin C.S. Wong, Paul K.S. Chan

**Affiliations:** ^1^Centre for Health Behaviours Research, School of Public Health and Primary Care, The Chinese University of Hong Kong, Hong Kong, China.; ^2^School of Public Health and Primary Care, The Chinese University of Hong Kong, Hong Kong, China.; ^3^Department of Microbiology, Faculty of Medicine, The Chinese University of Hong Kong, Hong Kong, China.

**Keywords:** COVID-19 Vaccination, Behavioral Intention, Effectiveness, Safety, Cost, Hong Kong

## Abstract

**Background:** The prevalence of coronavirus disease 2019 (COVID-19) vaccination is very critical in controlling COVID-19. This study mainly aimed to (1) investigate behavioral intentions of COVID-19 vaccination under various specific scenarios, and (2) associated factors of the afore-mentioned vaccination intentions.

**Methods:** A random anonymous telephone survey interviewed 450 Chinese adults from September 16-30, 2020 in Hong Kong, China. Nine scenarios of behavioral intentions of COVID-19 vaccinations were measured combining effectiveness (80% versus 50%), safety (rare versus common mild side effect), and cost (free versus HK$ 500).

**Results:** The prevalence of behavioral intentions of COVID-19 vaccination under the 9 specific scenarios was very low and varied greatly (4.2% to 38.0%). The prospective countries of manufacture also influenced vaccination intention (eg, Japan: 55.8% vs China: 31.1%). Only 13.1% intended to take up COVID-19 vaccination at the soonest upon its availability. The attributes of effectiveness and side effect influenced vaccination intention most. Positively associated factors of behavioral intentions of COVID-19 vaccination included trust/satisfaction toward the government, exposure to positive social media information about COVID-19 vaccines, descriptive norms, perceived impact on the pandemic, perceived duration of protectiveness, and life satisfaction.

**Conclusion:** Intention of COVID-19 vaccination was low in the Hong Kong general population, especially among younger people, females, and single people. Health promotion is warranted to enhance the intention. The significant factors identified in this study may be considered when designing such health promotion. Future research is required to confirm the findings in other countries. Such studies should pay attention to the specific context of cost, safety, and effectiveness, which would lead to different responses in the level of behavioral intention of COVID-19 vaccination (BICV).

## Background

**Key Messages**

** Implications for policy makers**

- The prevalence of behavioral intentions of coronavirus disease 2019 (COVID-19) vaccination was relatively low and varied greatly according to the effectiveness, side effect, and cost of COVID-19 vaccines in Hong Kong, China.
- Only a small portion of respondents considered taking immediate COVID-19 vaccination upon vaccines’ availability; the majority showed wait-and-see or reluctant attitude.
- Prospective countries of manufacturers of COVID-19 vaccines affected COVID-19 vaccination intentions greatly.
- Attributes of effectiveness and side effects had significant influences on COVID-19 vaccination.
- A wide range of determinants of COVID-19 vaccination was identified, including factors related to government, social media, COVID-19 perceptions, and mental health.

** Implications for the public**

Coronavirus disease 2019 (COVID-19) vaccines are crucial in controlling the COVID-19 pandemic globally; its coverage rate is important. In Hong Kong, China, the prevalence of behavioral intentions of COVID-19 vaccination was relatively low and varied greatly according to the vaccines’ effectiveness, side effects, and cost. The results showed that manufacturing countries influenced the vaccination intentions, and respondents cared most about vaccines’ effectiveness and side effects. Relative to a small proportion of respondents showing the intention of immediate vaccination upon vaccines’ availability, the majority showed hesitancy and even reluctance. The afore-mentioned COVID-19 vaccination intentions were associated with a number of factors, including trust/satisfaction toward government, exposure to related social media information, social norms, perceived impacts of vaccines on controlling the pandemic, perceived duration of protectiveness, and overall life satisfaction. The findings suggest that great efforts are needed to improve prevalence of COVID-19 vaccination, from vaccine development to policy-making to health promotion.

The coronavirus disease 2019 (COVID-19) has globally accumulated 35 347 404 cases and 1 039 406 deaths (6/10/2020).^1^ Recently, the pandemic has resurged in many countries. In most countries, the current measures seem inadequate in halting the pandemic, which has comprehensively damaged people’s lives and mental health.^2,3^ While herd immunity without vaccination is not foreseeable,^4^ the development of COVID-19 vaccines is seen as the ultimate hope.^4^ Is it a panacea? Ten of the 169 COVID-19 vaccine candidates have entered Phase III clinical trials.^5^ Various governments are pre-ordering vaccines under testing in huge amounts.^6^ Such expeditions heighten people’s expectation, but also introduce uncertainties about effectiveness, safety, cost, distribution equity, and compromised scientific standard.^7,8^

 Coverage of COVID-19 vaccination is very crucial. Vaccination hesitancy is problematic.^9^ It is likely to occur for a number of reasons. First, the COVID-19 vaccines have been developed in unprecedentedly short period of time. Even among experts, there are uncertainties about the length of immunity period and long-term side effects. Second, vaccine hesitancy is a universal problem; World Health Organization (WHO) described vaccine hesitancy as a global health challenge.^10^ The FDA sets the minimal acceptable effectiveness level of 50% for COVID-19 vaccines,^11^ which would require a very high population coverage to attain herd immunity (possibly >70%).^12^ One simulation study suggested that a vaccination coverage rate of 75% is required to control the COVID-19 pandemic, even the vaccines’ effectiveness reach 80%.^13^ In literature, some countries (eg, Malaysia, China, India, and Canada) reported very high prevalence of intention of vaccination (80.1%-94.3%)^14-17^; other countries (eg, Italy, the United States, European countries, and Israel) reported moderately high prevalence (57.6%-75%).^18-23^ There are gaps in knowledge about the factors of COVID-19 vaccination. Factors of influenza vaccination included perceptions toward influenza and related vaccines, interpersonal factors (eg, peer influences and social media), and psychological factors (eg, perceived stress).^24-26^ The limited literature on the factors of COVID-19 vaccination only looked at socio-demographics (eg, males, age, and educational level) and health beliefs (eg, perceived risk of COVID-19 and perceived efficacy of COVID-19 vaccines).^15,15,18-22,27^ Besides, such studies have pivotal limitations. (1) Interpretations/inferences are difficult as most of them were based on online snowball sampling^20,23^; none were population-based and only 2 involved random sampling.^15,18^ (2) Perceived effectiveness/safety/cost/country of manufacture affect vaccination intention. Yet, no study specified such contexts in the question on intention; the reference frames were hence blur. (3) The intentions of all these studies were not time-bounded, while many people indicating a vaccination intention may wait-and-see. (4) They did not investigated attributes affecting the decision process (eg, duration of protectiveness, report of severe side effect, and social media) and may have missed important factors of intention of vaccination (eg, trust toward the government, social media influences, mental health, and perceived impact). Improvements are warranted.

 The socio-ecological model postulates that determinants of health behaviors include those of structural (contextual), interpersonal, and individual levels. In this study, (1) structural (contextual) factors included satisfaction and trust toward the government, which were positively associated with preventive behaviors (eg, social distancing) during the COVID-19 pandemic^28,29^; (2) interpersonal-level factors included social media discussion and messages, which may affect people’s evaluation and performance of behaviors^29^; (3) individual level factors included cognitive factors derived from the theory of planned behaviors (TPB), which has been commonly used to understand determinants of behavioral intention of various health-related behaviors.^30^ It postulates that attitudes (perceived impact and perceived duration of protectiveness of COVID-19 vaccination in this case), subjective norm (descriptive norms in this case), and perceived behavioral control would affect behavioral intention.^30^ This study did not include perceived behavioral control, as the conditions (eg, availability, timing, cost, and priorities of COVID-19 vaccination) would largely be determined by the government; people might not know yet how much they could control COVID-19 vaccination at this “pre-vaccination stage.” (4) In addition, individual level factors of perceived risk, past behavior (influenza vaccination), and psychological factors (life satisfaction) were tested as such variables were significantly associated with preventive behaviors.^14,31,32^

 The present study was conducted in the general adult population in Hong Kong. Contextually, since February 2020, the Hong Kong government has implemented rather stringent prevention and control measures (eg, mandatory quarantines and contact tracing for suspicious cases, entry/exit restrictions, class suspensions, cancellation of large events, working from home policies, and gathering restriction of <10 persons), which have been under regular adjustments according to the changes in severity of the local pandemic. The use of facemasks and practice of good hand hygiene were almost universal (>95%).^33^ From February to mid-September 2020 (when the survey started), life in Hong Kong has been affected but remained more or less normal; there was no lockdown although there were periods that many people worked from home. On average, there were about 10 new cases per day and cumulatively 4984 cases and 102 deaths as of mid-September 2020. However, the prevalence of vaccination in the general public was not outstanding (eg, 43.9% to 45.8% for influenza vaccination and pneumococcal vaccination among people aged ≥65 years in the past 2 years^34^). Thus, in the context of relatively low COVID-19 infection rate, high levels of personal protection, relatively normal life, and relatively low past prevalence of vaccinations in the general population, the prevalence of COVID-19 vaccination is uncertain and warrants research.

 This study investigated the levels of (1) behavioral intention of COVID-19 vaccination (BICV) during the first 6 months since its availability to the general public in Hong Kong under 9 scenarios of specific cost/effectiveness/safety combinations and 5 scenarios of free vaccination according to the manufacturing country, (2) attitudes toward timing of vaccination, and (3) attributes influencing the participants’ vaccination decision. As previous studies reported significant sex and age differences in healthcare service utilization in general,^35,36^ and preventive behaviors related to COVID-19 including BICV in particular,^15,21,31,37^ we also investigated sex and age differences in the levels of (1) to (3) afore-mentioned.It also investigated factors of BICV and attitude for vaccination at the soonest upon availability (ie, socio-demographics, 3 aspects of trust/satisfaction toward the government, positive social media messages, and perceptions about COVID-19 vaccination).

## Methods

###  Study Design

 An anonymous random telephone survey (n = 450) of 15-20 minutes was conducted among Chinese speaking Hong Kong residents (aged ≥18) during September 16-30, 2020, and between 6-10:30 pm to avoid over-sampling non-working individuals. The telephone survey was based on landline phones. Simple random sampling was conducted, with landline numbers being randomly drawn from the most updated residential telephone directory. Unanswered telephone calls were given at least 3 attempts. To maintain the statistically independence nature of the sampled participants, only one household member was selected from each valid household. When there were multiple eligible household members, a simple random sampling procedure (the ‘next birthday rule’) was exercised, ie, the household member whose birthday was closest to the survey date was invited to participate in the study. Such random selection method within household has been commonly used in many published telephone survey studies.^38,39^ Appointments were made if necessary. Experienced interviewers briefed the participants about the study and sought verbal informed consent. The interviewers were requested to cross-check whether they have followed the briefing and informed consent process described in their training manual upon completion of each interview, and then signed a form to confirm such had been done. No incentives were given to the participants. Participants could quit any time. The response rate was 51.4%. The design has been used in many published studies.^40,41^ Ethics approval was obtained from the corresponding author’s affiliated institution.

###  Measures

*Background factors *included sex, age, educational level, marital status, whether having children under 18 years old, employment status, and self-reported chronic disease status (ie, whether having any of the listed chronic diseases, including hypertension, diabetes, chronic pulmonary diseases, myocardial infarction, myocardial failure, cerebrovascular diseases, ulcerative diseases, hepatic diseases, and tumors).

####  Intention of COVID-19 Vaccination

1. BICV: The level of intention (1 = definitely not to 5 = definitely yes) was asked under 9 specific scenario combinations (S1-S9): If the Hong Kong government provides free COVID-19 vaccination to the public within 6 months since its availability in Hong Kong, how likely would you take up the vaccination if it is under the following situations? (*a*) if the vaccines have 80% effectiveness while mild side effects (MSE) rarely occur (S1), (*b*) if the vaccines have 80% effectiveness while MSE commonly occur (S3), (*c*) if the vaccines have 50% effectiveness while MSE rarely occur (S5), (*d*) if the vaccines have 50% effectiveness while MSE commonly occur (S7). The questions were repeated for the other four scenarios (S2, S4, S6, S8) that involved a fee of HK$ 500, instead of free vaccination. The ninth scenario was: if the vaccines have 80% effectiveness while severe side effects rarely occur. The 5-point response options were recoded into binary categories (“will likely/definitely taking up COVID-19 vaccination” versus “else”) for estimation of prevalence of BICV and being used as the dependent variables of the logistic regression analysis. The recoded binary variables allow for comparisons with numerous studies that used similar dichotomizations to define outcomes of behavioral intention of vaccination.^14,15,19^ It also has clearer public health implications for understanding how many people might take up the vaccination.
2. Behavioral intention to take upfree COVID-19 vaccination according to the manufacturing countries (within 6 months since its availability in Hong Kong), including (*i*) Japan, (*ii*) Europe/the United States, (*iii*) mainland China, (*iv*) Russia, and (*v*) other countries (1 = definitely not to 5 = definitely yes).

####  Attitudes Toward COVID-19 Vaccination

1. Attitudes toward timing of COVID-19 vaccination: One item assessed such attitudes (5-point response options: ‘at the soonest,’ ‘wait till having a good comprehension about the vaccines’ effectiveness/safety,’ ‘as late as possible,’ and ‘try not to/definitely not’).
2. Attributes influencing vaccination decision (0 = no influence at all to 10 = extremely strong influences): (*i*) effectiveness, (*ii*) duration of protectiveness, (*iii*) side effect, (*iv*) cost, (*v*) country of manufacture, (*vi*) convenience, (*vii*) expert recommendation, (*viii*) evaluations from social media, (*ix*), support from family members, and (*x*) report of severe side effects.
3. Perceived availability of free vaccination and equity: The 3 items assessed (*i*) whether the government should provide free vaccination to all permanent residents (yes/no response options), (*ii*) perceived likelihoods that the government would provide free COVID-19 vaccination to the participant in the coming year (1 = definitely not to 5 = definitely yes), and (*iii*) perceived chance of inequity in obtaining free COVID-19 vaccination (1 = definitely not to 5 = definitely yes).
4. Willingness to pay: “what is the maximum amount that you would like to pay for COVID-19 vaccination” (3 points: ≤100 HK$, 101-300 HK$, >300 HK$).

####  External Factors of Vaccination Intentions

1. Trust/satisfaction toward the government: The 3 separated items were overall trust toward the government (1 = strongly mistrust to 5 = strongly trust), trust regarding governmental COVID-19 measures (1 = strongly mistrust to 5 = strongly trust), and overall satisfaction toward the government (1 = strongly dissatisfied to 5 = strongly satisfied).
2. Frequency of exposure to positive social media messages about COVID-19 vaccines: “How often did you obtain positive information about COIVD-19 vaccination from the social media (1 = extremely infrequent to 5 = extremely frequent)?”

####  Internal (Personal-Level Cognitive) Factors of Vaccination Intentions

1. Descriptive norms (2 items): perceived percentages (in increments of 10%) of the general public and acquaintances taking up COVID-19 vaccination during the six months since its availability.
2. Perceived impact of COVID-19 vaccination: “With COVID-19 vaccination, what is the likelihood of controlling COVID-19 locally?” (1 = very low to 5 = very high).
3. Perceived duration of protectiveness of COVID-19 vaccines (<1 year/ ≥1 year/don’t know).
4. Perceived risk: “What is the chance of you contracting COVID-19 in the coming year (1 = very low to 5 = very high)?”
5. Life satisfaction: “I am satisfied with my life” (1 = extremely disagree to 7 = extremely agree).
6. Influenza vaccination in the past 12 months (yes/no).

###  Data Analysis

 The sample size planning was conducted by using the logistic regression module in the PASS 11.0. Assuming the prevalence of BICV ranged from 20% to 90%, the sample size of 450 would have the smallest detectable odds ratio (OR) of 1.39 to 1.55 (power of 0.80 and alpha of 0.05, two-sided). Thus, the study would have a good statistical power of at least 0.80 for associations with OR≥1.39/1.55. The sample size is thus adequate.

 McNemar, *t* test, analysis of variance, and chi-square tests were performed to test group differences. As mentioned, BICV was recoded into a binary variable (“likely/definitely yes” versus “else”). The analysis was done in 2 stages. We firstly looked at the socio-demographic differences in BICV and attitude of vaccination at the soonest, as those factors have important implications on health promotion that are independent of those related to the socio-ecological factors (eg, cognitions, social media influence, and trust). In the second stage, we investigated the socio-ecological factors adjusting for the socio-demographic factors, as the socio-ecological factors may also be associated with the background factors and may thus confound the associations between the socio-ecological factors and BICV and attitude of vaccination at the soonest. Crude odds ratios (ORc), adjusted odds ratios (ORa), and their 95% confidence intervals (CI) were derived. SPSS 21.0 was used for data analysis. Statistical significance was defined as *P*< .05 (two-tailed).

## Results

###  Background Characteristics 

 Over half of the participants were females (68.9%), aged >35 years (86.0%), had not attended colleges (69.1%) and were currently married (70.2%); 11.8% had children aged <18; 1/3 worked full-time (34.2%) and suffered from chronic diseases (eg, hypertension and diabetes) (32.7%) (Table 1).

**Table 1:** Descriptive Statistics

	**n**	**%**
**Background Factors**		
Gender		
kFemale	310	68.9
Male	140	31.1
Age groups		
18-35	63	14.0
36-65	243	54.0
>65	144	32.0
Educational level		
<College	311	69.1
≥College	138	30.7
Missing data	1	0.2
Current marital status		
Married	316	70.2
Single	92	20.4
Else	42	9.3
Having children under 18		
No	397	88.2
Yes	53	11.8
Employment status		
Full-time	154	34.2
Retired	137	30.4
Housewives	116	25.8
Else	43	9.6
Chronic diseases status		
No	302	67.1
Yes	147	32.7
Don’t know	1	0.2
**External Factors of BICV**		
Overall trust toward the government		
Very strong mistrust	54	12.0
Mistrust	78	17.3
Neutral	128	28.4
Trust	172	38.2
Very strong trust	9	2.0
Don’t know	9	2.0
Trust toward governmental measures for controlling COVID-19		
Very strong mistrust	37	8.2
Mistrust	92	20.4
Neutral	153	34.0
Trust	153	34.0
Very strong trust	15	3.3
Overall satisfaction toward the government		
Very strong dissatisfaction	53	11.8
Dissatisfaction	79	17.6
Neutral	151	33.6
Satisfaction	152	33.8
Very strong satisfaction	8	1.8
Don’t know	7	1.6
Frequency of exposure to positive social media messages about COVID-19 vaccines		
Extremely infrequent	43	9.6
Quite infrequent	90	20.0
Average	214	47.6
Quite frequent	101	22.4
Extremely frequent	1	0.2
Don’t know	1	0.2
**Attitude About Timing of COVID-19 Vaccination**		
At the soonest	59	13.1
Wait for clarification about the vaccines’ effectiveness and safety	307	68.2
At late as possible	46	10.2
Try not to	18	4.0
Definitely not	20	4.4
**Perceptions About Availability of Free Vaccination**		
Whether the government should provide free vaccination to every permanent resident		
No	15	3.3
Yes	435	96.7
Perceived chance that the government would provide free vaccination in the coming year		
Definitely not	13	2.9
Probably not	37	8.2
Half-half	152	33.8
Probably yes	185	41.1
Definitely yes	57	12.7
Don’t know	6	1.3
Perceived chance of inequity regarding free COVID-19 vaccination		
Definitely not	66	14.7
Probably not	80	17.8
Half-half	114	25.3
Probably yes	95	21.1
Definitely yes	51	11.3
Don’t know	44	9.8
The maximum amount of cost that participants were willing to pay (HKD)		
≤100	83	18.4
101-300	190	42.2
>300	126	27.9
Don’t know	51	11.3
**Internal Factors of BICV**		
Perceived effectiveness of COVID-19 vaccines on controlling COVID-19 in Hong Kong		
Very low	5	1.1
Quite low	35	7.8
Average	168	37.3
Quite strong	213	47.3
Very strong	22	4.9
Don’t know	7	1.6
Perceived duration of protectiveness of the COVID-19 vaccine (year)		
<1	141	31.3
≥1	141	31.3
Don’t know	168	37.3
Perceived risk		
Very low	36	8.0
Quite low	221	49.1
Moderate	143	31.8
Quite high	45	10.0
Very high	5	1.1
Influenza vaccination in the past 12 months		
No	334	74.2
Yes	115	25.6
Don’t know	1	0.2

Abbreviations: COVID-19, coronavirus disease 2019; BICV: Behavioral intention for likely/definitely taking up COVID-19 vaccination during the first 6 months since its availability to the general public in Hong Kong.

###  The Prevalence of BICV Under the 9 Scenarios 

**Figure 1:**
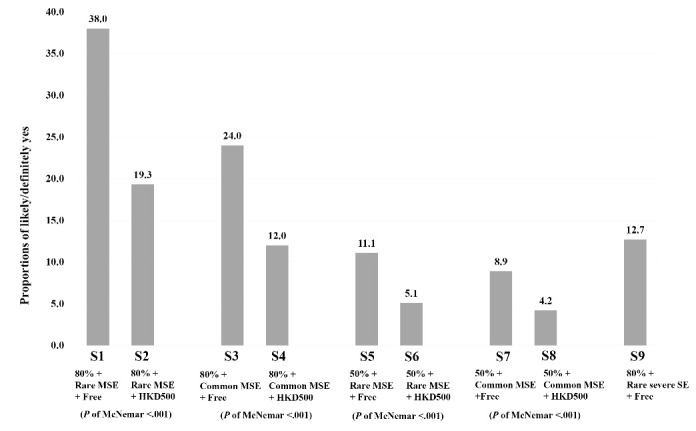
BICV Under 9 Scenarios of Effectiveness, Safety, and Charge Combinations (for All Participants). Abbreviation: BICV, behavioral intention for COVID-19 vaccination.

 Among all the participants (Figure 1), the highest prevalence of the binary variable of BICV (likely/definitely yes) was 38.0% under the ‘best’ scenario (S1: free/80% effectiveness/rare MSE) (definitely yes: 4.0%; likely: 34.0%; not sure: 19.8%; unlikely: 10.2%; definitely not: 32.0%). Two scenarios, both involved 80% effectiveness (free/common MSE and HK$ 500/rare MSE), had prevalence of about 20%. If the vaccination costs HK$ 500 *or* has 50% effectiveness* or* has rare severe side effect (S4-S9), the prevalence fell short of 13%. A cost of HK$ 500 would almost half the prevalence of intention in all the S1-S8 scenarios (*P*< .001; McNemar test). The standardized prevalence of BICV (weighted by sex and age census data) of S1 to S9 was compared to the crude rates (in brackets): S1: 34.4% (38%), S2: 19.4% (19.3%), S3: 20.2% (24.0%), S4: 11.7% (12.0%), S5: 11.5% (11.1%), S6:.2% (5.1%), S7: 8.7% (8.9%), S8: 5.1% (4.2%), and S9:11.1% (12.7%).

**Figure 2:**
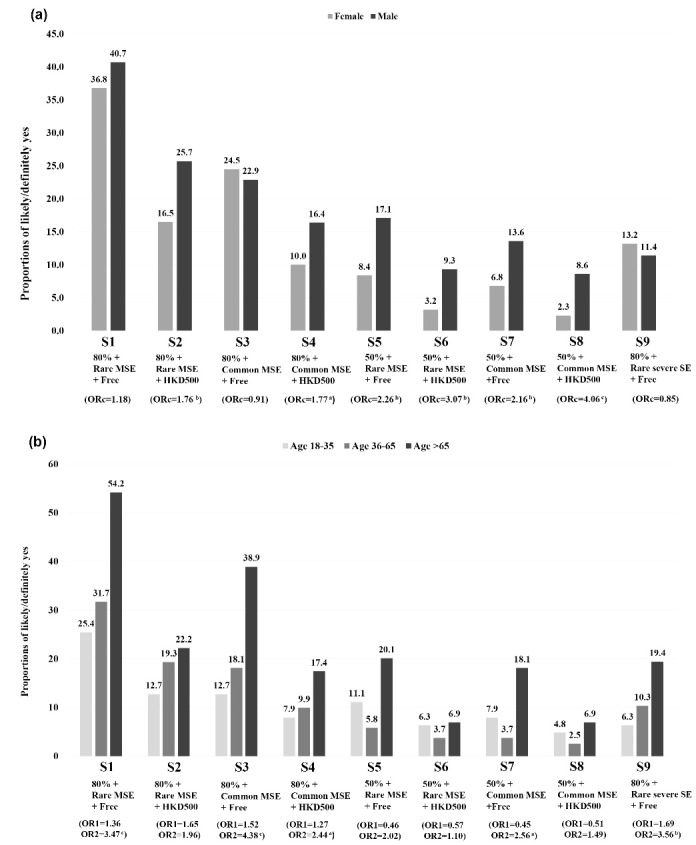
**
(a)** BICV Under 9 Scenarios by Sex (reference group = female). **(b)** BICV under 9 scenarios by age [OR1: Age 36-65 versus Age 18-35 (reference group); OR2: Age >65 versus age 18-35 (reference group); ^a^.05 < *P *<.10; ^b^*P *< .05; ^c^*P *< .01; ^d^*P *< .001. Abbreviations: BICV, behavioral intention for COVID-19 vaccination; ORc, crude odds ratio].

 In Figure 2a, males expressed higher BICV than females in two of the five 80% effectiveness scenarios that involved a HK$ 500 cost (S2, S4) and all the four 50% effectiveness scenarios (S5-S8) (ORc ranged from 1.76 to 4.06, *P*< .05). In Figure 2b, the oldest group showed significantly higher prevalence of BICV than the youngest group in the 3 scenarios of free vaccination and 80% effectiveness (S1, S3, and S9: ORc = 3.56 to 4.38; *P*< .05). In the ‘best’ scenario (S1), for instance, the prevalence of the youngest versus oldest groups was 54.2% versus 25.4% (ORc = 3.47, 95% CI: 1.80-6.69).

###  Prevalence of Intention of Free COVID-19 Vaccination According to the Manufacturing Country

 The prevalence was higher if the free vaccines were manufactured in Japan (55.8%) and the United States/Europe (52.0%), and lower if manufactured in mainland China (31.1%), Russia (32.2%), and other countries (27.8%). Higher age but not sex was in general associated with such intentions (Table S1, Supplementary file 1). Besides, only 6.9% of the participants would likely/definitely take up COVID-19 vaccination in mainland China if the vaccine were available there but not in Hong Kong (data not tabulated).

###  Attitudes About Timing of COVID-19 Vaccination

 Only 13.1% of all the participants would take up COVID-19 vaccination at the soonest; 68.2% would wait for clarification about the vaccines’ effectiveness/safety; the rest either wish to take up vaccination as late as possible (10.2%) or avoid taking it up (8.4%). The sex difference was non-significant. Older people were more likely than younger people to incline toward vaccination at the soonest, and were less likely to wait-and-see or avoid vaccination (chi-square test; *P*< .001; data were not tabulated).

###  Specific Attributes Influencing Decision on COVID-19 Vaccination 

 The mean (SD) of the levels of influences of such attributes were: *( i ) side effect *[8.3 (1.8)]* (ii) effectiveness *[8.2 (1.8)],* (iii) report of severe side effects *[8.1 (1.9)],* (iv) duration of protection against COVID-19 *[7.0 (2.3)], *(v) experts’ recommendation *[6.7 (1.9)],* (vi) cost *[6.7 (2.0) for price], *(vii) country of manufacture *[6.7 (2.5)],* (viii) support from family members *[6.7 (2.4)], *(ix) convenience *[6.1 (2.3)],and* (x) evaluation from social media *[5.7 (2.3)] (ranges of the item responses: 0-10; mid-point: 5.5). The sex differences were statistically non-significant for 9 of the ten attributes, except for family support [mean (SD) for male: 6.3 (2.6); female: 6.9 (2.3);* P*< .007]. Age was negatively associated with the attributes of effectiveness [mean (SD): aged 18-35 = 8.3 (1.6); aged 36-65 = 8.6 (1.4); aged >65 = 7.9 (2.2); *P*= .001], side effect [mean (SD): aged 18-35 = 8.3 (1.5); aged 36-65 = 8.4 (1.5); aged >65 = 7.9 (2.3); *P*= .035], and country of manufacture [mean (SD): aged 18-35 = 7.6 (2.0); aged 36-65 = 7.1 (2.3); aged >65 = 5.7 (2.8); *P*< .001] (Table S2, Supplementary file 1).

###  Perceptions About Availability of Free Vaccination 

 Almost all (96.7%) perceived that the government should provide free vaccination to every permanent resident; 53.8% perceived that the government would provide free vaccines to him/her in the coming year. About 1/3 (32.3%) perceived future inequity regarding free COVID-19 vaccination; older people tended to think so; sex was non-significant. Only 27.9% was willing to pay over HK$ 300 for COVID-19 vaccination (Table 1).

###  Factors of BICV Under the 9 Scenarios

####  Level of the Potential Factors 

 Over one-third indicated trust/strong (overall) trust toward the government and trust specific to COVID-19 measures (42.3% and 37.3%, respectively); 35.6% was satisfied with the government. About 1/4 (22.6%) were frequently/very frequently exposed to positive social media messages about COVID-19 vaccines. About half (52.2%) believed that the vaccines would have strong/very strong impacts on controlling the pandemic locally; 31.3% perceived the vaccines would be protective for ≥1 year (don’t know: 37.3%); 25.6% had taken up influenza vaccination (past 12 months) (Table 1). It was estimated that on average, 40%-50% and 30%-40% of the general public and the acquaintance would take up COVID-19 vaccination, respectively. The mean score (SD) of life satisfaction was 4.7 (1.2) (range: 1 to 7) (Data are not tabulated).

####  Background Factors of BICV

Table 2 and Table S3 (Supplementary file 1) described 7 out of the 9 scenarios (except for S6 and S8 that had very low prevalence of BICV): (1) Positively associated factors (*P*< .05) included employment status (retirement and housewife) (S1, S3, S5, S7, S9) and chronic disease status (S1, S3, S5, S7, S9); (2) negatively associated factors (*P*< .05) included higher educational level (S1 and S3) and being single (S1); (3) having children aged <18 showed no significant associations (*P*> .05) under all scenarios.

**Table 2:** Background Factors of BICV Under Scenario 1 and Inclination Toward Immediate COVID-19 Vaccination

**Variables**	**S1: 80% + Rare MSE + Free (Likely/Definitely Yes)**	**Vaccination at the Soonest**
	**ORc (95% CI)**	**ORc (95% CI)**
Educational level		
<College	Ref = 1.0	Ref = 1.0
≥College	0.54 (0.35-0.84)^c^	0.27 (0.12-0.60)^c^
Missing data	NA	NA
Current marital status		
Married	Ref = 1.0	Ref = 1.0
Single	0.50 (0.30-0.85)^b^	0.29 (0.10-0.83)^b^
Else	1.66 (0.87-3.17)	2.54 (1.21-5.34)^b^
Having children under 18		
No	Ref = 1.0	Ref = 1.0
Yes	0.55 (0.29-1.04)^a^	0.23 (0.06-0.99)^b^
Employment status		
Full-time	Ref = 1.0	Ref = 1.0
Retired	2.54 (1.57-4.14)^d^	3.44 (1.73-6.86)^d^
Housewives	1.70 (1.02-2.83)^b^	1.37 (0.61-3.08)
Else	1.25 (0.60-2.58)	NA
Chronic disease status		
No	Ref = 1.0	Ref = 1.0
Yes	3.15 (2.09-4.75)^d^	3.93 (2.23-6.94)^d^
Don’t know	NA	NA

Abbreviations: BICV, behavioral intention for COVID-19 vaccination; ORc, crude odds ratio; Ref: reference group; DV, dependent variable; NA, not applicable; MSE, mild side effects; SE, side effect. Note. Scenario 1: COVID-19 vaccine of 80% effectiveness, rare MSE, and free. ^a^.05 < *P *<.10; ^b^*P *< .05; ^c^*P *< .01; ^d^*P *< .001.

####  External and Internal Factors of BICV

 Adjusted for all the background factors, the significance/directions of the external/internal factors were highly consistent across the 7 scenarios (Table S4, Supplementary file 1). We thus use S1 as an illustration (Table 3). Significant external factors included (1) overall trust toward the government (ORa = 8.22), (2) trust toward the government’s COVID-19 policies (ORa = 7.39), (3) satisfaction with the government (ORa = 8.97), and (4) frequency of exposure to positive social media messages about COVID-19 vaccination (ORa = 3.02). Significant internal (person-level cognitive) factors included: (1) perceived percentages of the public and acquaintances intending to take up COVID-19 vaccination within six months since its availability (ORa = 1.34 and 1.48, respectively), (2) perceived impact of the vaccines in controlling the local COVID-19 pandemic (ORa = 3.56), (3) perceived vaccines’ protectiveness ≥1 year (ORa = 2.65), (4) life satisfaction (ORa = 1.43), and (5) influenza vaccination (past 12 months) (ORa = 2.11). Unexpectedly, perceived risk was significantly but negatively associated with BICV in some scenarios (S3, S4, and S9).

**Table 3:** Adjusted Analysis on the Associations Between External/Internal Factors and BICV Under Scenario 1 and Immediate Vaccination

**Variables**	**S1: 80% + Rare MSE + Free ** **(Likely/Definitely Yes)**	**Vaccination at the ** **Soonest**
	**ORa (95% CI)**	**ORa (95% CI)**
**External Factors of BICV**		
Overall trust toward government		
Very strong mistrust/mistrust	Ref = 1.0	Ref = 1.0
Neutral	3.99 (1.99-7.99)^d^	2.93 (0.70-12.33)
Trust/very strong trust	8.22 (4.12-16.41)^d^	9.39 (2.40-36.76)^c^
Don’t know	4.17 (0.87-19.99)^a^	5.71 (0.42-77.47)
Trust toward governmental measures in controlling COVID-19		
Very strong mistrust/mistrust	Ref = 1.0	Ref = 1.0
Neutral	2.73 (1.40-5.33)^c^	3.69 (0.84-16.21)^a^
Trust/very strong trust	7.39 (3.74-14.61)^d^	11.97 (2.75-52.13)^c^
Overall satisfaction with government		
Very strong dissatisfaction/Dissatisfaction	Ref = 1.0	Ref = 1.0
Neutral	4.22 (2.16-8.25)^d^	6.16 (1.23-30.87)^b^
Satisfaction/very strong satisfaction	8.97 (4.41-18.24)^d^	17.43 (3.42-88.85)^c^
Don’t know	3.54 (0.58-21.60)	10.75 (0.67-172.44)^a^
Frequency of exposure to positive social media messages about COVID-19 vaccines		
Extremely/quite infrequent	Ref = 1.0	Ref = 1.0
Average	0.96 (0.58-1.57)	0.58 (0.28-1.20)
Extremely/quite frequent	3.02 (1.69-5.39)^d^	1.35 (0.61-3.02)
Don’t know	NA	NA
**Internal Factors of BICV**		
Descriptive norms		
Perceived level of vaccination among Hong Kong citizens^e^	1.34 (1.18-1.53)^d^	1.15 (0.95-1.40)
Perceived level of vaccination among acquaintances^e^	1.48 (1.32-1.66)^d^	1.41 (1.22-1.63)^d^
Perceived impact of COVID-19 vaccine on controlling COVID-19 in Hong Kong		
Very low/quite low	Ref = 1.0	Ref = 1.0
Moderate	0.97 (0.41-2.32)	1.31 (0.43-3.95)
Quite strong/very strong	3.56 (1.54-8.20)^c^	2.04 (0.16-25.98)
Don’t know	0.72(0.07-7.18)	
Perceived duration of effectiveness of the COVID-19 vaccine		
<1 year	Ref = 1.0	Ref = 1.0
≥1 year	2.65 (1.58-4.45)^d^	2.83 (1.14-7.02)^b^
Don’t know	0.80 (0.47-1.36)	3.06 (1.28-7.36)^b^
Perceived risk		
Low/very low	Ref = 1.0	Ref = 1.0
Moderate	1.38 (0.88-2.17)	1.45 (0.77-2.75)
High/very high	0.60 (0.29-1.21)	0.24 (0.07-0.79)^b^
Life satisfaction^e^	1.43 (1.15-1.78)^c^	1.43 (1.00-2.04)^b^
Influenza vaccination in the past 12 months		
No	Ref = 1.0	Ref = 1.0
Yes	2.11 (1.29-3.45)^c^	3.50 (1.79-6.81)^d^
Don’t know	NA	NA

Abbreviations: BICV, behavioral intention for COVID-19 vaccination; ORa, adjusted odds ratio (the models were adjusted for sex, age groups, educational level, current marital status, having children under 18, employment status, and chronic disease status); Ref: reference group; DV, dependent variable; NA, not applicable; MSE, mild side effects; SE, side effect. *Note*. Scenario 1: COVID-19 vaccine of 80% effectiveness, rare MSE, and free. ^a^.05 < *P *<.10; ^b^*P *< .05; ^c^*P *< .01; ^d^*P *< .001; ^e^ Those who answered “don’t know” and refused to answer were excluded from the analysis.

###  Factors Associated With Attitude of Vaccination at the Soonest 

 Retirement and chronic disease status were positively associated with such an attitude; higher educational level, single marital status, and having children aged <18 showed negative associations. The significance/directions of the ORa of the external/internal factors were very similar to those under the 7 scenarios of BICV. Consistent to the scenarios of S3, S4, and S9, perceived risk was significantly and negatively associated with the attitude of vaccination at the soonest (Table 3 and Table S4).

## Discussion

 A wide range of prevalence of BICV (4.2%-38%) was observed under the 9 ‘combination scenarios’ of cost/ effectiveness/safety (6-month since availability). It alerts readers about ambiguities of previously reported prevalence of vaccination intention, which was based on unconditional measures, as the participants’ responses in such studies involved heterogeneous assumptions about cost/performance/safety/timeframe. The potentially misleading shortcoming could not be adjusted statistically. Similar research needs to be context specific.

 The prevalence, even under the ideal scenario (S1: free/80% effective/rare MSE) was low (38%) and dropped to ≤13% in the more realistic scenarios of 50% effectiveness or a cost of HK$ 500. The prevalence of actual behaviors is usually lower than that of intention. Thus, it is very doubtful whether Hong Kong could achieve herd immunity within a few months, a year, or more. It seems over-optimistic to expect that life would soon return to normal.

 The Hong Kong prevalence was among the lowest (4%-20%) across countries if we consider scenarios other than the best one. Hong Kong’s prevalence of vaccination intention seems much lower than that of India, Malaysia, Italy, Israel, the United States, and mainland China (about 60%-90%). Hong Kong has in general exercised good control over COVID-19. During the pandemic period, there were very often <10 new cases/day (102 deaths), good testing/quarantine plans, and good hand hygiene and almost universal face-mask use in public areas.^33^ It is plausible that Hong Kong people might rely less on vaccination as a means of protection against COVID-19 and hence showed lower vaccination intention. Cross-country studies are warranted to test the contention.

 It is warranted to promote COVID-19 in the Hong Kong general population. Special efforts are required to promote COVID-19 vaccination among subgroups reporting very low prevalence of BICV, such as females, younger people, and single people, as these groups showed very high levels of vaccination hesitancy. Even the younger groups might have milder consequences related to COVID-19, high prevalence of COVID-19 vaccination in these groups is still required to achieve herd immunity and community protection. They were more concerned about effectiveness and safety than older people; evidence-based promotion approach may be useful.

 Older people and those with chronic diseases were vulnerable groups in terms of severity of COVID-19 infection^42^ and should be prioritized for COVID-19 vaccination. The results show that they were more likely than others to show BICV. However, even for the older group (>65), about half of them did not indicate BICV; health promotion is thus still required. To promote COVID-19 vaccination in these groups, logistics for COVID-19 vaccination needs to be age-friendly. For instance, special arrangements may be required for those living in nursing home and/or suffering from serious disease conditions. It is interesting that older people’s decision on COVID-19 vaccination was less influenced by effectiveness, safety, and the manufacturing country of COVID-19 vaccines. It is plausible that such influences might have been over-ridden by their concerns about severity (eg, high fatality rate among chronic disease patients). It is also plausible that some older people might have lower education levels and/or possess low literacy about vaccine effectiveness and safety. Special attention should be given to these vulnerable groups.

 Even Hong Kong is affluent, vaccination cost would half the prevalence of BICV. The willingness to pay was only moderate and comparable to the US$ 30 reported in Malaysia.^14^ The downturns in economy may have reduced the willingness-to-pay. No study has investigated the impact of cost on COVID-19 vaccination intention. Almost all participants believed that the government should provide free vaccination to every permanent resident but only about half believed that a free vaccination would be provided for him/her in the coming year. Vulnerable subgroups (eg, older people and those with chronic diseases) should be prioritized to receive vaccination. While priority needs to be set, equity may become a concern, as only 32.5% believed that inequity would not emerge. Such pressing issues need to be addressed, especially in places like Hong Kong where trust of the government is low.

 No published study has looked at how the vaccines’ country of manufacture would affect intention of COVID-19 vaccination, which was much higher if the vaccines were produced in Japan/Europe/US than if they were produced in mainland China/Russia/other countries. Despite the promising development of the Chinese COVID-19 vaccines announced in scientific communities and the high possibility that China will become one of the major global vaccine producers, it is astonishing that 90% of the participants would not take up COVID-19 vaccination in mainland China, even if there were no supply in Hong Kong. Politicization might contribute to such low confidence, beyond scientific evaluations from credible authorities. Health promotion about rational scientific appraisals of COVID-19 vaccines is necessary. Another implication is that some governments, including that of Hong Kong, might be unable to order all doses of vaccines from a single country. Allocation of vaccines produced by several countries to the public is likely a controversial and political task.

 While some politicians are extremely eager to launch COVID-19 vaccination promptly and the Hong Kong government has started pre-ordering vaccines that have not passed Phase III trials, the general public would not rush, as only 13% would take up the vaccines at the soonest (wait-and-see: 70%; reluctance: 20%). Given such attitudes and the low level of intention and concerns about country of manufacture, governments need to consider expiry dates and possibilities of more effective second generation COVID-19 vaccines before stocking up first generation ones.

 As mentioned, health promotion is required to boost the intention to take up safe and effective COVID-19 vaccines. The findings confirm that effectiveness and side effect affected BICV most, and highlight other considerations that should be factored into health promotion (eg, duration of protectiveness/cost/country of manufacture/expert recommendation/convenience/family support/evaluations of social media). Significant structural (political) factors (eg, trust toward the government and political views) were reported in 3 US and Canadian studies.^16,22,27^ Mistrust and dissatisfaction toward the government in general and specific to the COVID-19 were severe in Hong Kong,^43^ and have become a global trend (eg, the Unites States and Europe).^44,45^ Such attitudes reduced vaccination intention as indicated by this study’s strong ORs. It is urgently warranted to strengthen social capital and research on its impact on BICV.

 Social media’s positive messages were associated with BICV and should be incorporated into health promotion (eg, scientific evidence, number of vaccinations, and personal testimonials). Health promotion should modify personal-level perceptions significantly associated with BICV. For the first time, perceived duration of the vaccines’ protectiveness was found to be associated with BICV. It fits in the attitudinal construct of the TPB, and is similar to the construct of perceived benefits of the Health Belief Model, which has been applied to study acceptance of COVID-19 vaccines.^46^ The estimated proportions of the public/acquaintance having BICV (descriptive norms) were associated with BICV; descriptive norm is part of the subjective norm of the TPB^46^; such norms can be instilled into the general population. Furthermore, according to the transactional model of stress and coping, life satisfaction would result in positive coping^47^ and healthy behaviors^48,49^; it was positively associated with BICV. The pandemic has threatened population mental health,^2^ which had reduced protective behaviors against COVID-19.^49,50^ It is important to maintain the public’s life satisfaction during the pandemic.

 Against our hypothesis, negative instead of positive associations between this variable and BICV was observed. It is plausible that those having an intention of COVID-19 vaccination and those who showed an attitude of vaccination at the soonest might believe that they would take up and be protected by COVID-19 vaccination, and thus would have lower likelihoods of contract COVID-19 in the coming year, instead of the hypothesized situation that those who perceived high (immediate) risk would be more motivated to show BICV. The cross-sectional study design did not allow us to distinguish between cause and effect.

 The study has the strength of being one of the few studies with random sampling on BICV. Our ‘forecast’ facilitates planning. The response rate of <60% may introduce selection bias, although it is comparable to other published studies using telephone surveys.^40,41^ We have compared the demographic composition (ie, sex, age, and educational level) of our studied population against that of the 2018 Hong Kong census data. While the sample’s educational level was comparable to that of census data, there were some differences. (1) The sample’s male group was underrepresented (31.1% versus 45.0% of the census); (2) the 18-35 age group was underrepresented (14.0% versus 22.8% of the census) and the >65 age group was overrepresented (32.0% versus 21.2% of the census). However, the standardized prevalence of BICV weighted by sex and age census data were only slightly different from the sample estimates. Thus, the differences might be acceptable.

 There are other limitations: (1) Self-reported data may introduce selection and reporting biases. (2) We studied intentions that might not be translated into real behaviors. (3) Due to the length of the question items, we did not explain to the participants how rare was rare, nor about the specific types of mild and severe side effects, as there are many possibilities. (4) We did not randomize the sequence of the 9 scenarios (S1-S9) as there were too many combinations and might confuse the participants if the 9 scenarios were presented in a random order. An ordering effect might exist. We asked the questions of the 5 free vaccination scenarios first (in the order of S1, S3, S5, S7, then S9), followed by those of the 4 self-paid scenarios (in the order of S2, S4, S6, and S8). Such an order might have inflated the prevalence of behavioral intention of self-paid COVID-19 vaccination if those who had reported willingness to take up free vaccination would feel less socially desirable to switch their responses to no intention of self-paid vaccination. (5) Some scenarios had not been considered (eg, number of doses required) and the cost of HK$ 500 was arbitrarily set; it is also a limitation that we did not specify the setting when asking the participants about attitude toward timing of COVID-19 vaccination. (6) Some of the scales were constructed for this study as validated scales were unavailable. (7) The study may have missed important factors (eg, self-efficacy, perceived barriers, and coping styles). (8) The cross-sectional study cannot establish causality.

## Conclusion

 In sum, the low and wide range of prevalence of BICV reminds us of the importance in using specific ‘combinations scenarios’ approaches to understand BICV. This study has several implications. (1) Mistrust with the government and the vaccines’ manufacturing countries needs to be rectified. (2) Transparent/stringent/credible international standards need to be upheld. (3) Science needs to be separated from politics. (4) The governments may not rush toward launching large-scale vaccination for any reasons until warranted scientific evidence and public acceptance are established. (5) Cost would reduce vaccinations greatly; willingness to pay may only be moderate. (6) Equity is a potential issue; efforts need to protect financially disadvantaged groups. Considering COVID-19 vaccination record as one of the facilitators for travel arrangements (eg, Hong Kong to mainland/foreign countries) may increase the vaccination rate but needs thorough discussion of the pros and cons. Regarding research, international comparative studies and longitudinal surveillance on changes in BICV under different scenarios prior to and after launching COVID-19 vaccines are strongly recommended to inform policy formulation and adjustments. Health promotion is required and may take this study’s findings into account.

## Acknowledgement

 We would like to thank Dr. Meiqi Xin for her assistance in literature search. We would also like to thank all the participants for their contributions.

## Ethical issues

 The study was approved by the Survey and Behavioral Research Ethics Committee of the Chinese University of Hong Kong (No. SBRE-20-034).

## Competing interests

 Authors declare that they have no competing interests.

## Authors’ contributions

 Conceptualization: JTFL and YQY; Methodology: YQY, JTFL; Investigation: MMCL; Software: YQY; Formal analysis: YQY; Data curation: YQY; Validation: JTFL; Resources: JTFL; Writing-original draft: YQY and JTFL; Writing-review & editing: YQY, JTFL, MCSW, and PKSC; Supervision: JTFL; Funding acquisition: JTFL.

## Funding

 The study was supported by internal research funding of the Centre for Health Behaviours Research. The funding source has no role in this study.

## Supplementary files

Supplementary file 1 contains Tables S1-S4.
