# Supplementary material for: Understanding the Prevalence and Associated Factors of Behavioral Intention of COVID-19 Vaccination Under Specific Scenarios Combining Effectiveness, Safety, and Cost in the Hong Kong Chinese General Population
Source: Int J Health Policy Manag. 2021 Jan 9;11(7):1090–101. doi: 10.34172/ijhpm.2021.02 (PMC9808181; doi:10.34172/ijhpm.2021.02)
Supplement: Supplementary file 1 — contains Tables S1-S4. [file ijhpm-11-1090-s001.pdf]

Article title: Understanding the prevalence and associated factors of behavioral intention of COVID-19 vaccination under specific scenarios combining effectiveness, safety, and cost in the Hong Kong Chinese general population

Supplementary file 1: Supplementary tables of "Understanding the prevalence and associated factors of behavioral intention of COVID-19 vaccination under specific scenarios combining effectiveness, safety, and cost in the Hong Kong Chinese general population"

Table S1. Prevalence of intention of taking up free COVID-19 vaccination (likely/definitely yes) given the vaccines were manufactured in a specific country

	All	Sex		Age groups		
		Female	Male	18-35	36-65	>65
Country of manufacture	%	% ORc (Ref)	% ORc (95% CI)	% ORc (Ref)	% ORc (95% CI)	% ORc (95% CI)
Japan	55.8	54.8	57.9	46.0	56.4	59.0
		Ref=1.0	1.13 (0.76-1.69)	Ref=1.0	1.52 (0.87-2.64)	1.69 [a] (0.93-3.07)
The United States/Europe	52.0	51.3	53.6	42.9	49.8	59.7
		Ref=1.0	1.10 (0.74-1.64)	Ref=1.0	1.32 (0.76-2.31)	1.98 [b] (1.09-3.60)
Mainland China	31.1	29.7	34.3	9.5	26.3	48.6
		Ref=1.0	1.24 (0.81-1.89)	Ref=1.0	3.40 [c] (1.40-8.26)	8.99 [d] (3.65-22.16)
Russia	32.2	28.4	40.7	19.0	24.7	50.7
		Ref=1.0	1.73 [b] (1.14-2.63)	Ref=1.0	1.39 (0.70-2.79)	4.37 [d] (2.15-8.88)
Other countries	27.8	25.5	32.9	15.9	20.2	45.8
		Ref=1.0	1.43 (0.93-2.21)	Ref=1.0	1.34 (0.64-2.82)	4.49 [d] (2.12-9.50)

Note. COVID-19: coronavirus disease 2019; ORc: crude odds ratio; Ref: reference group; a, .05<*P*<.10; b, *P*<.05; c, *P*<.01; d, *P*<.001.

Table S2. Levels of vaccine-related attributes influencing whether taking up COVID-19 vaccination by sex and age

Variables	All	Sex			Age groups			
		Female	Male	*P* (t-test)	18-35	36-65	>65	*P* (ANOVA)
	Mean (SD)	Mean (SD)	Mean (SD)		Mean (SD)	Mean (SD)	Mean (SD)	
1) Side effect	8.3 (1.8)	8.4 (1.7)	8.2 (1.9)	.175	8.3 (1.6)	8.6 (1.4)	7.9 (2.2)	.001
2) Effectiveness	8.2(1.8)	8.22 (1.8)	8.24 (2.0)	.930	8.3 (1.5)	8.4 (1.5)	7.9 (2.3)	.035
3) Report on severe side effect	8.1 (1.9)	8.1 (1.8)	8.0 (2.2)	.629	8.3 (1.6)	8.1 (1.8)	7.9 (2.2)	.297
4) Duration of protectiveness against COVID-19	7.0 (2.3)	6.6 (2.6)	6.9 (2.4)	.055	6.9 (2.1)	7.0 (2.3)	6.6 (2.7)	.264
5) Expert recommendation	6.73(1.9)	6.8 (2.0)	6.7 (2.0)	.370	6.9 (2.1)	6.9 (1.9)	6.6 (2.2)	.233
6) Cost	6.72(2.0)	6.8 (2.0)	6.6 (2.2)	.412	6.3 (2.1)	6.7 (1.7)	6.9 (2.4)	.144
7) Manufacturer	6.71(2.5)	6.8 (2.5)	6.5 (2.7)	.340	7.6 (2.0)	7.1 (2.3)	5.7 (2.8)	<.001
7) Support from family members	6.71 (2.4)	6.9 (2.3)	6.3 (2.6)	.007	6.5 (2.2)	6.6 (2.3)	7.0 (2.6)	.136
9) Convenience	6.1 (2.3)	6.2 (2.2)	5.9 (2.3)	.229	6.1 (2.2)	6.1 (2.1)	6.2 (2.5)	.925
10) Evaluation from social media	5.7 (2.3)	5.8 (2.3)	5.5 (2.2)	.195	6.2 (2.0)	5.7 (2.3)	5.4 (2.4)	.064

Table S3. Background factors of behavioral intention of COVID-19 vaccination under various scenarios

Variables	S1: 80% + Rare MSE + Free (Likely/definitely yes)	S2: 80% + Rare MSE + HKD500 (Likely/definitely yes)	S3: 80% + Common MSE + Free (Likely /definitely yes)	S4: 80% + Common MSE + HKD500 (Likely /definitely yes)	S5: 50% + Rare MSE + Free (Likely/definitely yes)	S7: 50% + Common MSE + Free (Likely /definitely yes)	S9: 80% + Rare severe SE + Free (Likely/definitely yes)
	ORc (95% CI)	ORc (95% CI)	ORc (95% CI)	ORc (95% CI)	ORc (95% CI)	ORc (95% CI)	ORc (95% CI)
Educational level							
< College	Ref=1.0	Ref=1.0	Ref=1.0	Ref=1.0	Ref=1.0	Ref=1.0	Ref=1.0
≥ College	0.54 (0.35-0.84)[c]	0.73 (0.43-1.25)	0.54 (0.32-0.90)[b]	0.88 (0.47-1.65)	0.96 (0.51-1.83)	0.84 (0.41-1.74)	0.70 (0.37-1.33)
Missing data	NA	NA	NA	NA	NA	NA	NA
Current marital status							
Married	Ref=1.0	Ref=1.0	Ref=1.0	Ref=1.0	Ref=1.0	Ref=1.0	Ref=1.0
Single	0.50 (0.30-0.85)[b]	0.55 (0.28-1.06)[a]	0.54 (0.29-1.00) [a]	0.75 (0.35-1.61)	0.77 (0.34-1.71)	0.72 (0.29-1.79)	0.54 (0.23-1.24)
Else	1.66 (0.87-3.17)	0.73 (0.31-1.72)	1.67 (0.84-3.29)	0.93 (0.35-2.51)	1.61 (0.66-3.89)	1.71 (0.67-4.42)	1.53 (0.67-3.54)
Having children under 18							
No	Ref=1.0	Ref=1.0	Ref=1.0	Ref=1.0	Ref=1.0	Ref=1.0	Ref=1.0
Yes	0.55 (0.29-1.04) [a]	0.72 (0.32-1.58)	0.53 (0.24-1.16)	0.57 (0.20-1.64)	0.45 (0.13-1.49)	0.18 (0.02-1.31) [a]	0.38 (0.12-1.27)
Employment status							
Full-time	Ref=1.0	Ref=1.0	Ref=1.0	Ref=1.0	Ref=1.0	Ref=1.0	Ref=1.0
Retired	2.54 (1.57-4.14) [d]	1.26 (0.71-2.24)	2.43 (1.42-4.16)[b]	1.29 (0.66-2.51)	3.19(1.52-6.71) [c]	3.88 (1.68-8.95) [c]	1.32 (0.62-2.82)
Housewives	1.70 (1.02-2.83) [b]	1.24 (0.68-2.26)	1.37 (0.75-2.47)	0.82 (0.38-1.77)	0.71 (0.25-1.98)	0.65 (0.19-2.22)	2.34 (1.14-4.81) [b]
Else	1.25 (0.60-2.58)	0.46 (0.15-1.40)	0.59 (0.21-1.64)	0.35 (0.08-1.55)	2.11 (0.73-6.08)	1.87 (0.54-6.54)	1.32 (0.45-3.88)
Chronic disease status							
No	Ref=1.0	Ref=1.0	Ref=1.0	Ref=1.0	Ref=1.0	Ref=1.0	Ref=1.0
Yes	3.15(2.09-4.75)[d]	1.80 (1.11-2.91) [b]	3.12(1.99-4.88) [d]	2.29(1.29-4.07)[c]	3.00(1.65-5.45) [d]	3.11(1.61-6.03) [c]	1.46 (0.83-2.59)
Don't know	NA	NA	NA	NA	NA	NA	NA

Note. ORc, crude odds ratio; Ref, reference group; DV, dependent variable; NA, not applicable; MSE, mild side effect; SE, side effect; a, .05<*P*<.10; b, *P*<.05; c, *P*<.01; d, *P*<.001.

Table S4. Adjusted analysis for factors 4 of behavioral intentions of COVID-19 vaccination under various scenarios

Variables	S1: 80% + Rare MSE + Free (Likely /definitely yes)	S2: 80% + Rare MSE + HKD500 (Likely /definitely yes)	S3: 80% + Common MSE + Free (Likely /definitely yes)	S4: 80% + Common MSE + HKD500 (Likely /definitely yes)	S5: 50% + Rare MSE + Free (Likely /definitely yes)	S7: 50% + Common MSE + Free (Likely /definitely yes)	S9: 80% + Rare severe SE + Free (Likely /definitely yes)
	ORc (95% CI)	ORc (95% CI)	ORc (95% CI)	ORc (95% CI)	ORc (95% CI)	ORc (95% CI)	ORc (95% CI)
**<u>External factors of BICV</u>**							
1.Overall trust toward government							
Very strong	Ref=1.0	Ref=1.0	Ref=1.0	Ref=1.0	Ref=1.0	Ref=1.0	Ref=1.0
Neutral	3.99(1.99-7.99) [d]	2.42 (1.09-5.40) [b]	3.54 (1.53-8.17) [c]	3.34 (1.29-8.67) [b]	3.41(1.06-10.98) [b]	4.75(1.27-17.82) [b]	2.23 (0.84-5.92)
Trust/very strong trust	8.22(4.12-16.41) [d]	3.34 (1.53-7.30) [c]	6.15(2.69-14.03) [d]	2.26 (0.85-5.97)	5.31(1.67-16.83) [c]	4.76(1.24-18.31) [b]	2.51(0.96-6.55) [a]
Don't know	4.17 (0.87-19.99) [a]	4.20 (0.71-24.82)	1.51 (0.16-14.42)	NA	9.93 (1.27-77.33) [b]	4.79 (0.36-62.91)	1.88 (0.19-19.17)
2.Trust toward governmental							
Very strong	Ref=1.0	Ref=1.0	Ref=1.0	Ref=1.0	Ref=1.0	Ref=1.0	Ref=1.0
Neutral	2.73 (1.40-5.33) [c]	1.14 (0.50-2.59)	2.20 (0.96-5.06) [a]	1.72 (0.65-4.58)	1.09 (0.36-3.32)	1.38 (0.38-4.96)	1.26 (0.48-3.33)
Trust/very strong trust	7.39(3.74-14.61) [d]	3.63 (1.67-7.87) [c]	6.52(2.86-14.85) [d]	2.54 (0.97-6.64) [a]	3.64 (1.28-10.34) [b]	3.88(1.15-13.16) [b]	2.31 (0.91-5.86) [a]
3.Overall satisfaction with government							
Very strong	Ref=1.0	Ref=1.0	Ref=1.0	Ref=1.0	Ref=1.0	Ref=1.0	Ref=1.0
Neutral	4.22(2.16-8.25) [d]	2.28 (1.05-4.96) [b]	4.03 (1.74-9.33) [c]	3.41 (1.29-9.00) [b]	2.36 (0.78-7.12)	4.02(1.08-14.89) [b]	2.79 (1.04-7.49) [b]
Satisfaction/very strong	8.97(4.41-18.24) [d]	3.88 (1.73-8.68) [c]	7.72(3.23-18.41) [d]	3.03 (1.07-8.55) [b]	5.43(1.73-17.04) [c]	5.84(1.45-23.48) [b]	3.12 (1.12-8.66) [b]
Don't know	3.54 (0.58-21.60)	2.27 (0.23-22.36)	2.16 (0.21-21.81)	NA	4.81 (0.39-58.71)	6.12 (0.43-86.38)	2.67 (0.24-29.10)
4. Frequency of exposure to positive social media							
Extremely/quite infrequent	Ref=1.0	Ref=1.0	Ref=1.0	Ref=1.0	Ref=1.0	Ref=1.0	Ref=1.0
Average	0.96 (0.58-1.57)	1.48 (0.77-2.82)	0.99 (0.55-1.80)	0.63 (0.29-1.39)	1.34 (0.56-3.19)	1.89 (0.70-5.11)	1.82 (0.73-4.52)
Extremely/quite frequent	3.02(1.69-5.39) [d]	3.67(1.83-7.35) [d]	3.85 (2.02-7.33) [d]	2.74 (1.28-5.88)	4.39(1.82-10.62) [c]	4.89(1.76-13.59) [c]	7.61(3.05-18.96) [d]
Don't know	NA	NA	NA	NA	NA	NA	NA

**Internal factors of BICV**							
1.Descriptive norms							
Perceived level of vaccination among Hong Kong citizens[e]	1.34(1.18-1.53) [d]	1.48(1.27-1.73) [d]	1.39 (1.20-1.61) [d]	1.53(1.28-1.84) [d]	1.24 (1.03-1.50) [b]	1.33(1.08-1.64) [c]	1.34(1.13-1.58) [c]
Perceived level of vaccination among acquaintances[e]	1.48(1.32-1.66) [d]	1.32 (1.18-1.47) [d]	1.30 (1.17-1.45) [d]	1.28(1.12-1.45) [d]	1.41(1.22-1.62) [d]	1.47(1.25-1.72) [d]	1.26(1.10-1.43) [c]
2.Perceived impact of COVID-19 vaccine on controlling COVID-19 in Hong Kong							
Very low/quite low	Ref=1.0	Ref=1.0	Ref=1.0	Ref=1.0	Ref=1.0	Ref=1.0	Ref=1.0
Moderate	0.97 (0.41-2.32)	1.13 (0.30-4.22)	1.05 (0.35-3.09)	1.22 (0.25-5.96)	1.31 (0.26-6.55)	1.22 (0.24-6.23)	1.32 (0.41-4.22)
Quite high/very high	3.56 (1.54-8.20) [c]	4.42 (1.27-15.38) [b]	3.43 (1.24-9.51) [b]	3.79 (0.85-17.01) [a]	4.28 (0.94-19.43) [a]	3.03 (0.66-13.95)	0.82 (0.26-2.61)
Don't know	0.72(0.07-7.18)	NA	1.46 (0.14-15.66)	NA	NA	NA	NA
3.Perceived duration of protectiveness of the COVID-19 vaccine							
< 1 year	Ref=1.0	Ref=1.0	Ref=1.0	Ref=1.0	Ref=1.0	Ref=1.0	Ref=1.0
≥ 1 year	2.65(1.58-4.45) [d]	2.11 (1.15-3.87) [b]	6.28(3.21-12.27) [d]	4.09(1.74-9.61) [c]	2.32 (1.05-5.14) [b]	2.60(1.06-6.36)*	1.77 (0.89-3.51)
Don't know	0.80 (0.47-1.36)	0.83 (0.43-1.59)	1.99 (1.00-3.95) [a]	1.87 (0.76-4.60)	1.03 (0.44-2.40)	1.05 (0.41-2.72)	0.43(0.18-1.00) [a]
4.Perceived risk							
Low/very low	Ref=1.0	Ref=1.0	Ref=1.0	Ref=1.0	Ref=1.0	Ref=1.0	Ref=1.0
Moderate	1.38 (0.88-2.17)	0.88 (0.51-1.50)	1.04 (0.63-1.72)	0.71 (0.37-1.38)	1.90 (0.98-3.70) [a]	1.55 (0.75-3.22)	1.38 (0.75-2.54)
High/very high	0.60 (0.29-1.21)	0.50 (0.20-1.24)	0.25 (0.10-0.61) [c]	0.15 (0.03-0.68) [b]	NA	NA	0.22 (0.05-0.99) [b]
5. Life satisfaction[e]	1.43 (1.15-1.78) [c]	1.58(1.20-2.08) [c]	1.50 (1.16-1.94) [c]	1.48 (1.07-2.06) [b]	1.88 (1.24-2.85) [c]	1.90(1.20-3.00) [c]	1.35(1.00-1.83) [a]
6.Influenza vaccination in the past 12 months							
No	Ref=1.0	Ref=1.0	Ref=1.0	Ref=1.0	Ref=1.0	Ref=1.0	Ref=1.0
Yes	2.11 (1.29-3.45) [c]	1.86 (1.04-3.33) [b]	2.27 (1.34-3.83) [c]	2.20 (1.11-4.37) [b]	3.00 (1.50-5.99) [c]	2.77(1.29-5.91) [c]	2.56(1.30-5.04) [c]
Don't know	NA	NA	NA	NA	NA	NA	NA

Note. ORa, adjusted odds ratio (the models were adjusted for sex, age, educational level, current marital status, having children under 18, employment status, and chronic disease status); Ref, reference group; DV, dependent variable; NA, not applicable; MSE, mild side effect; SE, side effect. a, .05<*P*<.10; b, *P*<.05; c, *P*<.01; d, *P*<.001; e, those who answered "don't know" and refused to answer were excluded from the analysis.
